# Promoting Self-Efficacy of Individuals With Autism in Practicing Social Skills in the Workplace Using Virtual Reality and Physiological Sensors: Mixed Methods Study

**DOI:** 10.2196/52157

**Published:** 2024-01-11

**Authors:** Sung-In Kim, So-youn Jang, Taewan Kim, Bogoan Kim, Dayoung Jeong, Taehyung Noh, Mingon Jeong, Kaely Hall, Meelim Kim, Hee Jeong Yoo, Kyungsik Han, Hwajung Hong, Jennifer G Kim

**Affiliations:** 1 Department of Psychiatry Seoul National University Bundang Hospital Bundang Republic of Korea; 2 Georgia Institute of Technology Atlanta, GA United States; 3 Department of Industrial Design Korea Advanced Institute of Science and Technology Daejeon Republic of Korea; 4 Department of Data Science Hanyang University Seoul Republic of Korea; 5 Department of Artificial Intelligence Hanyang University Seoul Republic of Korea; 6 School of Interactive Computing Georgia Institute of Technology Atlanta, GA United States; 7 Department of Preventive Medicine Yonsei University College of Medicine Seoul Republic of Korea; 8 Herbert Wertheim School of Public Health and Human Longevity Science University of California San Diego San Diego, CA United States; 9 The Design Lab University of California San Diego San Diego, CA United States; 10 Center for Wireless & Population Health Systems Calit2's Qualcomm Institute University of California San Diego San Diego, CA United States; 11 Department of Psychiatry Seoul National University Bundang Hospital Seongnam Republic of Korea; 12 Department of Psychiatry Seoul National University College of Medicine Seoul Republic of Korea

**Keywords:** autism, virtual reality, workplace, self-efficacy, social skills, data reflection

## Abstract

**Background:**

Individuals with autism often experience heightened anxiety in workplace environments because of challenges in communication and sensory overload. As these experiences can result in negative self-image, promoting their self-efficacy in the workplace is crucial. Virtual reality (VR) systems have emerged as promising tools for enhancing the self-efficacy of individuals with autism in navigating social scenarios, aiding in the identification of anxiety-inducing situations, and preparing for real-world interactions. However, there is limited research exploring the potential of VR to enhance self-efficacy by facilitating an understanding of emotional and physiological states during social skills practice.

**Objective:**

This study aims to develop and evaluate a VR system that enabled users to experience simulated work-related social scenarios and reflect on their behavioral and physiological data through data visualizations. We intended to investigate how these data, combined with the simulations, can support individuals with autism in building their self-efficacy in social skills.

**Methods:**

We developed WorkplaceVR, a comprehensive VR system designed for engagement in simulated work-related social scenarios, supplemented with data-driven reflections of users’ behavioral and physiological responses. A within-subject deployment study was subsequently conducted with 14 young adults with autism to examine WorkplaceVR’s feasibility. A mixed methods approach was used, compassing pre- and postsystem use assessments of participants’ self-efficacy perceptions.

**Results:**

The study results revealed WorkplaceVR’s effectiveness in enhancing social skills and self-efficacy among individuals with autism. First, participants exhibited a statistically significant increase in perceived self-efficacy following their engagement with the VR system (*P*=.02). Second, thematic analysis of the interview data confirmed that the VR system and reflections on the data fostered increased self-awareness among participants about social situations that trigger their anxiety, as well as the behaviors they exhibit during anxious moments. This increased self-awareness prompted the participants to recollect their related experiences in the real world and articulate anxiety management strategies. Furthermore, the insights uncovered motivated participants to engage in self-advocacy, as they wanted to share the insights with others.

**Conclusions:**

This study highlights the potential of VR simulations enriched with physiological and behavioral sensing as a valuable tool for augmenting self-efficacy in workplace social interactions for individuals with autism. Data reflection facilitated by physiological sensors helped participants with autism become more self-aware of their emotions and behaviors, advocate for their characteristics, and develop positive self-beliefs.

## Introduction

### Background

Approximately 40% of individuals with autism experience anxiety because of difficulties in socializing, sensory sensitivities, and other factors [1-3]. Specifically, workplace environments can amplify anxiety in individuals with autism, such as sensory overload, communication barriers, and unplanned interactions [4-8] as these environments are often designed with neurotypical expectations in mind. When people with autism constantly encounter situations that elicit anxiety, it can cause them to have negative self-beliefs about their skills and performance, which can lower their self-confidence in the workplace [9]. Therefore, self-efficacy—the personal judgment or belief in one’s ability to succeed in prospective situations—is crucial for people with autism because it can help individuals approach challenging workplace experiences from a positive perspective, as opposed to focusing on failure [10,11].

Virtual reality (VR) interventions have demonstrated potential effectiveness in promoting the self-efficacy of people with autism, as they provide a safe yet realistic environment to master specific social skills by offering opportunities to repetitively practice them [12-18]. Although this exposure and repetition are useful for mastering skills, gaining a deeper understanding of specific social situations that trigger anxiety can better prepare users to effectively manage those situations in the real world. An individual’s understanding of anxiety-inducing situations and crafting self-beliefs that they have the capabilities to succeed in those situations align with 2 key constructs of the self-efficacy theory by Bandura [19,20]—physiological states and verbal persuasion. Notably, VR can also support physiological state awareness and verbal persuasion. It can identify the VR situations that prompt significant changes in a user’s physiological data, enabling the user to reflect on these experiences and formulate strategies to respond effectively [18]. This process can empower the development of self-efficacy [19]. However, existing VR interventions often limit the scope of self-efficacy to mastery experiences only [21] and fail to provide the extended theoretical rationale or background of self-efficacy on the design or outcomes of the system.

In this study, we expanded the current VR system by incorporating a feedback model in which the user’s behavioral and physiological sensor data can be reviewed immediately following the experience simulation, allowing for situational reflection.

Our research aimed to increase self-efficacy through a VR-based social skill training system for individuals with autism. Furthermore, we investigated how the facilitation of self-understanding through incorporating reflection of physiological and behavioral data immediately after the social skills practice can impact self-efficacy in preparation for real-life scenarios.

### Objectives

This study had 2 main objectives. First, we developed WorkplaceVR, a VR application that allows people with autism to engage in simulated work-related scenarios to help understand their performance through data-driven reflection, with users’ behavioral and physiological data collected during the VR experience. Second, we evaluated the feasibility of the WorkplaceVR by conducting a deployment study with 14 young adults with autism. Using a mixed methods approach, we investigated the changes in self-efficacy among users with autism using pre- and postsurvey questionnaires. In addition, we conducted interviews to identify how participants with autism use, expect, and encounter challenges in the VR experience.

## Methods

**Figure 1:**
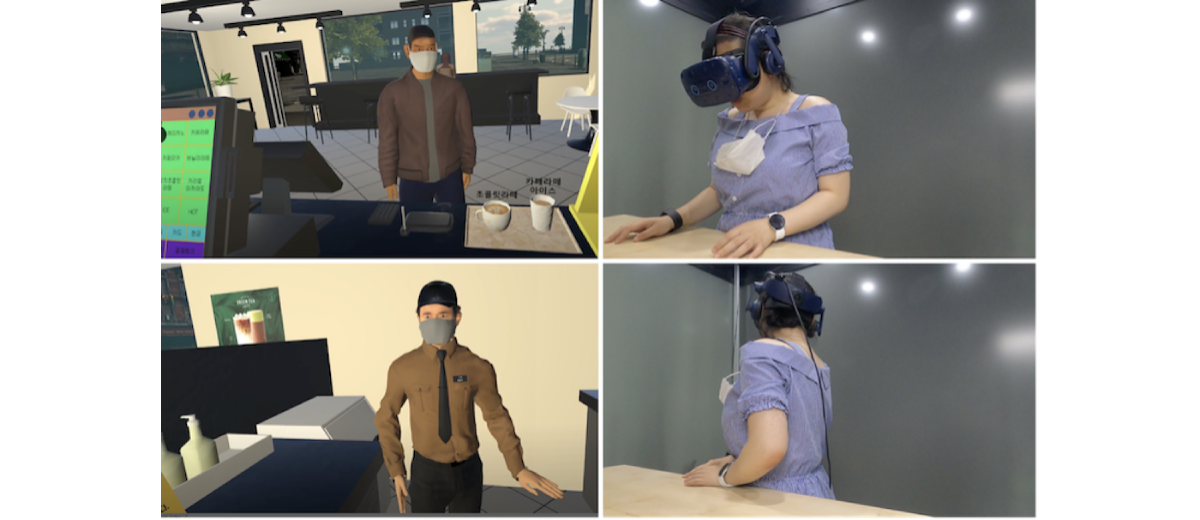
WorkplaceVR is a virtual reality–based system that offers immersive experiences of work-related social situations. With WorkplaceVR, our participants practiced their social skills in a simulated café environment in 2 basic-level and 2 advanced-level scenarios. Our findings highlighted that participants with autism were actively engaged in WorkplaceVR: placing their hands on the physical desk where the virtual counter was (top) and turning to face the virtual manager when conversing (bottom).

In this study, we designed and implemented WorkplaceVR, a VR-based system that offers simulations of work-related social situations and data-driven reflection of users’ behavioral and physiological responses (Figure 1).

### Phase 1: Development of the VR System (WorkplaceVR)

#### Inclusive and Iterative Design for the VR Program Development

Our user-centered design approach to developing the VR system is to ensure inclusivity; technology must empower a more diverse and inclusive society [22,23]. We aimed to build a safe and less stressful virtual environment for people with autism, where they can become familiar with the workplace environment and practice interacting with others without fear of failure. Moreover, with inclusive design in mind, we conducted an iterative design process to incorporate the voices of people with autism into the VR program.

We first conducted a preliminary study to draw design suggestions for the VR program [24]. To elicit end-user feedback, we created a 5-minute video prototype demonstrating the overarching concept and the use scenario of the WorkplaceVR program. Using the video prototype, we conducted semistructured interviews with 20 participants, including individuals with autism (employees, job seekers, etc), managers of companies where people with autism are currently working, psychiatrists, and professional counselors, to uncover the various needs of individuals with autism at workplaces. Through the interview results, we identified the following key design insights. First, the participants emphasized the importance of designing realistic VR scenarios to help users engage in the program by reflecting on their personal experiences or challenges at work. Second, the system should guide users to reflect on their emotions and behaviors. Finally, during the data reflection phase, participants with autism should be able to take control of expressing their thoughts and behaviors and build confidence.

With insights from the preliminary study, we developed our WorkplaceVR program (Figure 1). The system comprises 2 parts: the simulation VR scenario and the physiological data visualization interface. WorkplaceVR was developed using the Unity3D engine (Unity Technologies) and the SteamVR plug-in (Valve Inc) and runs on a Windows 10 (Microsoft) PC with an Intel Core i7, GeForce RTX 2070 graphics card, and 16 GB RAM. A head-mounted display (HMD), VIVE Pro Eye VR headset (HTC) [25], and Empatica E4 wristband (E4 band, Empatica Inc) [22] were worn by the users for viewing the virtual world and sensing physiological signals.

We conducted a pilot study with 4 neurotypical participants to assess the study protocol and identify any risks and challenges that users might experience in trying WorkplaceVR. The participants were introduced to the VR system, experienced WorkplaceVR using an HMD, and then asked to provide feedback on the overall experiences, including task difficulty and scenario length, visual components of the interface, and side effects of the VR experience if there were any (eg, motion sickness, headache, and visual disturbance). On the basis of participants’ suggestions, we added additional visual cues, such as a shining effect to objects (eg, the bill receipt) for users to quickly find and interact with in the 3D environment and an arrow user interaction icon to lead the eye to a specific direction or object to draw attention to specific objects or areas of interest when needed. Another problem raised by the users was regarding the test environment setup. To facilitate an environment where the users can feel safe and private, we placed a blackout curtain in front of the desk where they stood during the experiment.

#### WorkplaceVR: System Design

In this section, we elaborate on the features and design considerations of the VR system, WorkplaceVR, based on the self-efficacy theory by Bandura [19,20].

##### Designing VR Contents for Mastery Experience

VR can offer mastery experiences by simulating real-world scenarios that can engage users with autism to accomplish tasks related to workplace settings. To achieve this, our design approach focused on three key factors: (1) the inclusion of authentic work scenarios, (2) the integration of evidence-based social skills, and (3) the incorporation of multiple levels of difficulty within the scenarios.

1. Realistic work scenarios: we used café scenarios with work-related realistic visuals and auditory stimuli that provide a sense of surrounding in the immersive environment. For example, we designed café furniture and appliances (eg, a cash machine and a coffee machine) and instrumental background music to create a relaxing café atmosphere. Specifically, we selected a situation in which the barista must inform a customer that the drink they ordered is ready from among the situations that are required when working in a café (eg, greeting customers upon entry and taking customer drink orders).
2. Evidence-based social skills intervention: in specific café-based scenarios involving interactions between a barista and a customer, we incorporated evidence-based social skills (eg, active listening, initiating conversations, and not interrupting when someone else is talking) derived from interventions supported by previous research [26,27]. In addition, we included context-specific skills (such as informing customers about available options, verifying order accuracy, and problem-solving in unforeseen circumstances) sourced from the café service training manual [28,29].
3. Levels of scenarios: for the users to build self-efficacy, it is important to help them reduce anxiety and fear of failure by designing attainable goals that gradually increase in difficulty [19,30-32]. Thus, we designed 4 scenarios with 2 levels of difficulty: basic and advanced. The basic level requires the users to explore and interact with a simulated 3D environment to practice basic conversational skills as baristas. Following this, the users are involved in starting a conversation with a customer avatar by offering a polite greeting, taking orders, and serving beverages such as coffee. The 2 scenarios at this level have the same structure but different order details (number and types of beverages). When the users perform these tasks successfully, they are moved on to the advanced-level scenarios. At the advanced level, the scenario adds complications that arise within the conversation with the customer avatar (eg, a customer avatar claiming that their drink order is wrong).

##### Physiological Data and Visualization Interface

**Figure 2:**
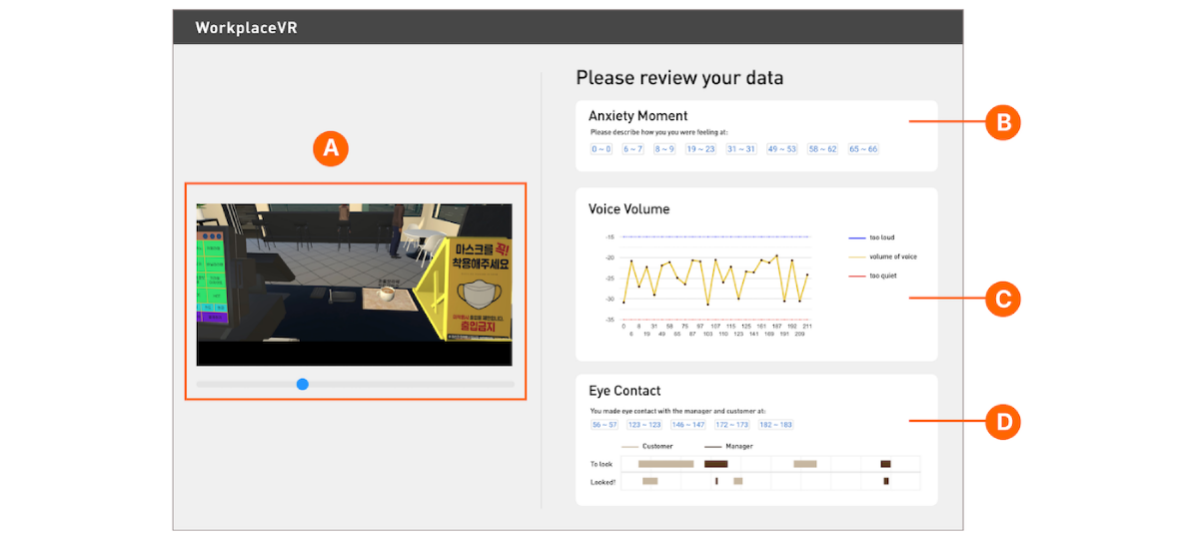
WorkplaceVR interface for data-driven reflection: consisting of (A) a video player that replays the user’s virtual reality experience and physiological or behavioral information visualizations that captured (B) anxiety moments, which are the time stamps that the Azure algorithm uses to detect indications of anxiety based on sensor data (eg, electrodermal activity and heart rate), and of user’s (C) volume of voice and (D) eye contact with the customer and manager avatar.

In the self-efficacy theory, Bandura [19,20] explains that people can shape their perceptions and beliefs about their capabilities by examining one’s physiological and emotional states. Therefore, one of our goals in designing the VR program was to help users better understand their emotions and behaviors by identifying their physiological states and behaviors in social situations [12,33]. We aimed to support people with autism to become more aware of their physiological and emotional states when they face stressful or anxiety-raising situations in the virtual work environment. Hence, we collected the users’ behavioral and physiological data during the VR intervention using the E4 band and HMD. We synchronized the time stamp information transmitted from E4 with the HMD time stamp information using the open-source framework Flask [34]. In addition, we presented a data visualization interface for users to understand and interpret their data along with their recorded performance videos, as illustrated in Figure 2. On the interface, we present (1) anxiety-related physiological measures (eg, temperature, electrodermal activity [EDA], and heart rate [HR]; Figure 2B); (2) changes in voice volume (Figure 2C); and (3) detection of eye contact (Figure 2D). The following explains how we defined, processed, and visualized each data type: anxiety moments, voice volume, and eye contact.

1. Anxiety moments: anxiety moments were defined using the time stamp on data collected when the sensors detected radical changes in the signal because of the users’ physiological reactions to the stimuli. We calculated anxiety moments using an anomaly detector provided by the Microsoft Azure machine learning algorithm [35]. This involved 5 sensor types—HR, EDA, temperature, interbeat intervals, and blood volume pulse—provided by the E4 band. We presented the anxiety moments on the interface with a time stamp range in seconds; each time stamp is linked to the exact time of the performance video. Users can click on a time range to watch their performance video on the left, as shown in Figure 2A.
2. Voice volume: voice data were collected using the microphone of the HMD. We then presented the voice volume level as a graph with time stamps to help users recognize changes in their voice tone and volume (Figure 2C).
3. Eye contact: to help participants understand their eye movements during their moments of anxiety, we used a region of interest (ROI), which is a specific area within an image or video selected for analysis. In our study, we set the faces of the customer and manager avatars as ROIs. We used the box collider of Unity3D and measured whether a user gazes at ROIs through Tobii G2oM [36], which is a machine learning algorithm that can accurately ascertain on which objects a user focuses. If the participant looked anywhere in that location, it was labeled as *seen (1)*; if they looked at another area, it was labeled *not seen (0)* in 1-second units. On the basis of the labeling results, we presented the seen labeled periods as a bar graph to help users understand how they make eye contact with people during VR scenarios, as presented in Figure 2D.

##### Verbal Persuasion Through User-Driven Insights

In this study, we focused on facilitating a data-driven reflection process to leverage verbal persuasion for users with autism to engage in the sense making of their physiological data. Our goal was to provide an opportunity for users with autism to navigate their physiological and behavioral data results, identify their own strengths and interests, and gain confidence in speaking about themselves. Thus, the participants were given an active role in interpreting their data and answering questions such as *What insights have you gained from the data about your characteristics or strengths?* and *What are your goals, taking data into account?*

### Phase 2: Implementation of the VR Program With Participants With Autism

We recruited participants with autism aged >16 years who are either currently employed or unemployed but plan on job searching in the near future. Our inclusion criteria for participants with autism were people who (1) have been diagnosed with autism; (2) can verbally articulate their thoughts, feelings, and experiences; (3) have no difficulties wearing an HMD for VR (eg, sickness or headaches while in VR or vision impairment, such as anisometropia); and (4) understand the study procedure and agree to participate. We posted the study posters to autism-related web-based communities on social networking services and web-based bulletin boards of autism-related institutions (eg, developmental disability social welfare centers and employment agencies for people with disability). In addition, we placed the flyers on the offline bulletin board of a child and adolescent psychiatry outpatient clinic in a hospital and a private counseling center for individuals with autism to outline the participant demographics.

### Ethical Considerations

To ensure ethical conduct, our study received approval from the Institutional Review Board of Seoul National University Bundang Hospital (institutional review board number: B-2202-736-302). As this study involved the collection of sensitive information (eg, physiological data) from participants, we informed the participants about the data collection process and obtained their informed consent before they participated in the study. The researchers explained the consent form to the participants with autism in easily understandable terms. We also clarified to the participants with autism that any personal information, VR data and sensor data collected during the research would be anonymized for analysis and securely discarded to protect their privacy. In addition, we offered a compensation of US $50 for the research participants.

### Measures

#### Perceived Self-Efficacy for VR Social Skill Training Scale

We developed the Perceived Self-Efficacy for VR Social Skill Training Scale (PSES-VR), an 8-item questionnaire with a 5-point Likert scale, to evaluate whether our VR intervention changed people’s beliefs regarding the self-efficacy of practicing social skills at the workplace. We developed this scale by modifying the Perceived Self-Efficacy questionnaire based on the theory of perceived self-efficacy by Bandura [37] and referencing existing scales, including the Perceived Social Self-Efficacy Scale [37], Perceived Improvement Scale [38], and Self-Efficacy Scale for Social Workers [39]. The questionnaires were revised to address the target social skills in the VR scenario. The 6 items consisted of communication skills required in general conversation situations. The 2 items evaluate whether participants respond appropriately to the situation required in the VR program scenario. Higher total scores on the items indicate better self-efficacy related to the social skills of the participants. The participants were asked to complete the same PSES-VR survey before the user study session and after the VR experience. All items of the PSES-VR are attached in Multimedia Appendix 1.

#### iGroup Presence Questionnaire

We used the iGroup Presence Questionnaire (IPQ), a 14-item questionnaire with a 7-point Likert scale, to investigate how users perceive a sense of presence of our VR system. The IPQ scale consists of 4 components: a general sense of being there (1 question), the sense of spatial presence (5 questions), involvement (4 questions), and experienced realism (4 questions), measuring the level of perceived presence during the VR experience [40]. Higher scores on the 4 components, as well as the total scores, indicate a better sense of presence in the VR system as perceived by the participants. All items of the IPQ are attached in Multimedia Appendix 1.

### Study Procedure

**Figure 3:**
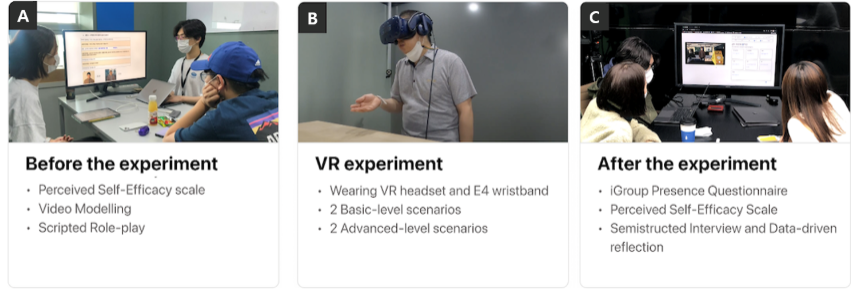
Overview of the study procedure: (A) before the experiment, (B) virtual reality (VR) experiment, and (C) after the experiment.

Our study procedure included three stages: (1) pre-experiment, (2) VR experiment, and (3) postexperiment reflection phase. Figure 3 presents a summary of the procedure.

#### Before the Experiment

To start, the researchers provided a brief overview of the study purpose and conducted a brief interview with participants, asking questions about their work-related experiences and previous experiences with VR. Next, the researchers introduced WorkplaceVR with explanations of the contents of the VR system and sensor devices that the participants should wear during the VR experiment. After that, the participants had a tutorial session to learn the 2 basic-level scenarios through video and scripted role-play.

#### VR Experiment

When the participants were ready for the VR experiment, they were asked to wear the E4 band, which is used for sensing the physiological signals, and to put on the VR headset. We informed the participants that they could stop at any time if they started experiencing motion sickness. After they wore the devices, we asked the participants to explore the café environment to adjust to the VR environment. Then, all participants performed the same 4 scenarios in the order of basic to advanced scenarios in VR for approximately 10 to 15 minutes.

#### After the Experiment

After the VR experience, we took a 5-minute break and conducted a data reflection on their VR performance with sensor data. In the data reflection process, participants were presented with various types of data related to their own anxiety moments, voice volume level, and eye contact while video recording their performance in VR using the interfaces and were asked to reflect on their performance based on these data. Here, we informed the participants that the data may not be accurate because of technical issues, so there can be errors or missing data in the interface. In the data reflection process, we asked the following to help participants reflect their data, such as “How was your performance in the VR scenarios?” “What were your strengths and challenges while performing the VR scenarios?” or “Did you find anything new about your behaviors while reviewing the data?” After the data reflection, we conducted a semistructured interview about the overall VR experience and data reflection. We asked participants how the VR content (eg, tasks and levels of scenarios, VR environment, and avatar design), data-driven reflection, and protocol (eg, watching a video and role-playing before the VR experiment) could be used to better understand their emotions and responses in potential workplace settings. We also asked questions to elicit feedback on the usefulness of data-driven reflection and its potential impact on their self-understanding of their behavior.

### Data Analysis

For qualitative data analysis, the interview data were transcribed, coded, and analyzed based on open coding and thematic analysis [41] using ATLAS.ti (version 7; Scientific Software Development GmbH). A total of 3 researchers individually read the interview transcripts and generated open codes that were discussed among the research team to identify patterns and build themes around VR experience and data-driven reflection regarding self-efficacy. The coding procedure was iterative; it concluded once the researchers agreed that the themes were consistent and a distinct set of themes surfaced. Interviews and surveys were conducted and documented in Korean, and analyses were performed in the same language. This ensured that the original responses were analyzed with the utmost precision. For the quotations used in the article, an English-Korean bilingual-speaking researcher initially translated the responses into English and then revised them through consultation with a proofreading expert.

For quantitative analysis, we analyzed the scale results (IPQ and PSES-VR) of the participants with autism. As a case-control group was not included in this study, it was difficult to confirm the statistical significance of the IPQ scale in identifying the presence of the VR system in participants with autism. For the IPQ scale, we calculated the mean and SD of the items corresponding to each of the 4 IPQ components and used it descriptively to analyze the VR presence of participants with autism. For the PSES-VR, we performed a paired 2-tailed *t* test to analyze whether there was a statistically significant change in perceived self-efficacy after using our VR system. For anxiety moments and eye contact sensor data, we conducted a 1-way ANOVA to analyze whether there was a statistically significant difference among the 4 scenarios in WorkplaceVR.

## Results

### Overview of the VR Experience

A total of individuals with autism participated in the study, including 2 women and 12 men, with an age range of 16 to 34 years. Table 1 shows the baseline characteristics of the participants.

There were no participants who reported difficulties such as motion sickness and headache during the VR experiments. Most (12/14, 86%) participants succeeded in completing the advanced-level scenarios without any support. In total, 2 participants (ND8 and ND13) completed the advanced-level scenario with minimal prompts from the researchers, such as guiding participants to find where the receipt was placed in the table.

**Table 1:** Demographic information about participants.

Code	Sex	Age (y)	Work experience (work period)
ND1	Male	23	Food service experience at a fast-food restaurant (1 y)
ND2	Male	32	Office worker (8 y)
ND3	Male	23	Undergraduate student or part-time job (assistant at a counseling center; 2 y)
ND4	Male	24	Part timer for a cleaning service and daily paid jobs (event staff; 4 y)
ND5	Female	22	Undergraduate student or daily paid jobs (PowerPoint presentation design; 1 mo)
ND6	Male	19	Undergraduate student and no working experience
ND7	Male	27	Undergraduate student or café barista (1 y)
ND8	Male	25	Remote worker (data entry in Excel) or designer (making web-based banners; 3 y)
ND9	Male	20	Undergraduate student and no working experience
ND10	Male	20	Undergraduate student or part-time work experience (warehouse loading truck job and restaurant server; 3 mo)
ND11	Male	20	Customer service agent (8 mo); staff at a central radio management service (4 mo)
ND12	Female	27	Part-time worker (pet care;1 y)
ND13	Male	33	Cleaning and maintenance (2 y)
ND14	Male	21	Freelancer (6 mo)

### Quantitative Assessment of the VR System Use

Our results showed a significant increase in the perceived self-efficacy of participants with autism (*P*=.02) before and after experiencing WorkplaceVR. In the IPQ result, the mean scores for “general presence” (or the “sense of being there”) and “experienced realism” were higher than the mean scores for the other 2 components (spatial presence and involvement). Table 2 presents the results of the questionnaires.

Table 3 presents the physiological sensor data of the participants in the 4 VR scenarios.

Our hypothesis was that participants with autism would experience more anxiety moments in the advanced scenarios. Consistent with the hypothesis, the participants had more anxiety moments in the advanced scenarios than in the basic scenarios (*P*<.001). This result shows that our physiological sensing data can effectively act as a proxy indicator for anxiety experienced by participants with autism. This is because individuals with autism tend to encounter elevated levels of anxiety in unexpected situations, such as the more advanced scenarios that we introduced.

**Table 2:** Results of the Self-Efficacy Scale and System Evaluation.

Scales		Values, mean (SD)	*P* value
**PSES-VR^a^**			.02^b^
	Pre	21.86 (7.33)	
	Post	39.07 (7.52)	
**IPQ^c^**			N/A^d^
	General presence	4.07 (1.86)	
	Spatial presence	3.87 (0.59)	
	Involvement	3.71 (0.79)	
	Experienced realism	3.89 (1.34)	

^a^PSES-VR: Perceived Self-Efficacy for VR Social Skill Training Scale.

^b^There was a significant increase in the perceived self-efficacy of participants with autism before and after their experience with WorkplaceVR.

^c^IPQ: iGroup Presence Questionnaire.

^d^N/A: not applicable.

**Table 3:** Physiological data of participants at each scenario.

Sensor data	Basic scenarios		Advanced scenarios		*P* value
	First	Second	First	Second	
Anxiety moments^a^ (number of points), mean (SD)	2.43 (3.56)	3.21 (3.36)	9.43 (4.96)	4.29 (2.70)	<.001^b^

^a^Anxiety moments are defined as the timestamps detected by the Azure algorithm’s anomaly detector when sensor signals (eg, electrodermal activity and heart rate) display fluctuations in response to users’ physiological reactions to stimuli.

^b^There were significantly more anxiety moments in the advanced scenarios than the basic scenarios.

### Qualitative Analysis of the VR System’s Impact on the Self-Efficacy

In this study, qualitative data analysis revealed 3 major themes that correspond to 3 of the 4 constructs of the self-efficacy theory by Bandura: physiological responses, verbal persuasion considered as encouragements, and mastery experience [19]. Below, we report how these findings can shed light on the feasibility of using the WorkplaceVR system to promote self-efficacy.

#### Impact of the VR Program on Self-Efficacy of Participants With Autism

##### Self-Awareness of Physiological and Emotional State Through Data Reflection

In the data reflection process, we provided an interface for visualizing the sensor data (ie, anxiety moments, voice volume level, and eye contact) to help the participants better recognize their emotions and behaviors in relation to their VR experiences. All our participants reviewed whether the presented data accurately reflected their emotions and behaviors. While reviewing their physiological signals (eg, HR, IBI, and EDA) presented by the VR system, the participants explained what they were feeling, thinking, or doing. For example, ND6 noticed that his HR increased when he was experiencing tense situations in VR and reported that he was aware of the stressors and reactions, drawing from his prior experiences:

I know that my heartbeat increases when I am in situations that make me anxious or nervous. I remember my heart beating so fast on the night of June 29th. There was also thunder that night [...] It was like the nervous feeling I get when I check my grades.

ND6

In addition, participants noted that the physiological data visualization provided valuable insight into their emotional states that may have otherwise been difficult to discern:

I don’t think I felt bodily sensations like stress or anxiety during the VR experience. But now, looking at the physiological data, I am convinced I was anxious in these situations [pointing at the “anxiety moments”].

ND12

Moreover, the participants recognized and described the patterns of when and why they felt anxious in certain situations. By watching the recorded video of their performance at “anxiety moments,” participants actively described when they were most stressed or anxious while carrying out the simulated workplace tasks (eg, when a customer avatar approaches or when a customer avatar makes a sudden request to the user). For instance, ND2 had 3 events marked as “anxiety moments,” all of which were related to tasks where the user was required to start a conversation with the customer avatar. After playing back the recorded videos for all 3 events, ND2 reported that the data well represented his characters and that he usually becomes anxious when he has to initiate conversations with people at work:

Oh, I’m quite sensitive sometimes, especially before I start talking, because I feel like I have to say something important. I also tremble when I start talking or see someone for the first time.

ND2

By reflecting on their physiological and emotional states and behaviors in advanced scenarios, it enabled participants to recollect their related experiences in the real world and talk about goals to tame anxiety. For example, after reflecting on anxious moments, ND6 discussed ways in which he can improve his comfort in similar situations in the future:

I don’t want to panic again when a problem arises. For example, I shouldn’t be too stressed about my exam results. Whether it be a good or bad result, what’s important is accepting the result and looking beyond it.

ND6

ND4 realized that he almost always becomes exhausted after meeting other people:

*When I come home after meeting someone, I get so tired and have a headache. I always wonderedwhat the reason was. Now that I think of it, I think it was because I got so nervous and tense in social situations.* [ND4]

In addition, some participants suggested that having more diverse data (eg, facial expressions, body movements, and gestures) could help them better reflect on and understand their behaviors:

It would be helpful to see how I make gestures and move my body because it is something that is hard to notice in real life. Having an observation camera might work.

ND7

##### Verbal Persuasion Through Self-Expression in Data Reflection

We found that the data-driven reflection process of our system can help participants with autism better understand themselves and build positive self-beliefs about who they are and what they can achieve. This opportunity allowed participants with autism to engage in verbal persuasion experiences of self-efficacy [42,43], which means that they could speak about their own strengths and interests and gain confidence in expressing themselves:

I was worried that I am not usually good at making eye contact. But seeing the data, I am surprised to realize that I am, in fact, doing quite well on this. I’m feeling more confident about myself.

ND10

In addition, some participants reported that they wanted to use behavioral and physiological data to explain the strengths and characteristics they recently discovered to others so they could express their thoughts or arguments more clearly. For example, ND12 reported that she feels misunderstood by people when they have the wrong impressions or ideas about her. She proposed ways to use the data to effectively communicate her opinions to decrease potential conflicts with others:

I often find myself in a state of persistent frustration when communicating with others. I believe that people do not truly understand or accept my thoughts or feelings. [...] However, with the physiological sensor data I currently possess, I am confident that I can convince others.

ND12

##### Mastery Experience Through Realistic VR Scenarios

Our interview results suggested that participants with autism experienced a sense of mastery while using the VR program. For example, the participants with autism reported that the successful completion of realistic workplace interpersonal situations in a VR environment boosted their confidence in handling similar situations in the real world:

You have to taste the fruits of success in order to gain confidence and not be afraid of failure. I felt this was an important factor for me to go on to the advanced level because failure can have a huge damaging effect.

ND4

This mastery experience was also supported by our VR system design that gradually increases the level of task difficulty from the basic-level scenarios to help users respond to unexpected situations with confidence:

Although it was new, it didn’t deviate too much from what I already experienced, so I could handle it.

ND14

Consistent with the IPQ result, the participants also reported a high level of presence in WorkplaceVR. The participants explained that this was because our VR program provided high-fidelity simulation embodying realistic visual content (eg, “Face Mask Required” signs on the walls of the virtual café considering COVID-19 pandemic situations) and immersive scenarios where they could have a naturalistic social interaction with virtual customers and colleagues (eg, using gestures to communicate and giving receipts to customers). For example, notable observations indicating high engagement included participants attempting tasks such as making coffee or using the cash machine, even though these actions were not part of the assigned tasks (Figure 1). The lifelike experiences participants experienced during our VR program could have played a role in cultivating a sense of mastery.

## Discussion

### Principal Findings

#### Overview

This study demonstrated that the VR program, which enables individuals with autism to experience work-related social scenarios and reflect on their VR practice through physiological and behavioral data visualization, can significantly increase the individuals’ perceived self-efficacy in practicing social skills within a workplace context. The interview data further showed how the data reflection of VR practice can influence self-efficacy. The user-driven data review practice allowed individuals with autism to reflect on their physiological data, that is, by promoting self-awareness of their emotions, gaining insights into their real-world behaviors that they were unaware of, and self-advocating their characteristics to others based on their data. In particular, participants could understand when and why they feel anxious, enabling them to proactively devise strategies for self-comfort in anticipation of similar anxiety-provoking situations. Moreover, the increased self-awareness about the underlying causes of their anxiety and related behaviors motivated them to communicate their experiences and advocate for their needs with others.

#### Promoting Self-Efficacy by Promoting Self-Awareness About Physiological States

We found that the physiological data reflection helped individuals better understand their emotional responses. This increased self-awareness that participants with autism obtained through our VR system motivated them to take their learnings from reflection in the real world to better explain and advocate for themselves to others. Therefore, in this section, we discuss how the self-reflection interface should be designed to present physiological and behavioral data in a way that encourages individuals to reflect on their experiences.

According to Bandura [19], recognizing and managing one’s own emotions and physiological states is essential in promoting self-efficacy, as this affects people’s decision-making process and performance. In line with the theory, our study found that behavioral and physiological sensor data (eg, eye contact, voice volume, EDA, and HR) could be used to support people with autism to become aware of their emotional states and, in turn, establish strategies to respond effectively to intense emotions. The participants became aware of their current affect state by mapping their physiological responses on the interface and sometimes wanted to examine data in depth to improve their self-understanding (eg, facial expression, standing posture, and hand gestures). Specifically, reflecting on the physiological data taken during the VR experience while watching the playback of the sessions allowed participants with autism to revisit how they felt and behaved in VR situations in specific moments that heightened their anxiety. This reflection reminded them of similar situations that induce anxiety in their daily lives, such as when they had to initiate conversations or when a conversational partner is approaching them. This increased self-awareness about their emotional responses—why they were anxious or nervous at specific times—further allowed participants with autism to make a resolution: how they might manage their emotional reactions in everyday lives.

Moreover, we found that the data reflection allowed participants with autism to gain insights about their real-world behaviors that they were unaware of (eg, “Why I was anxious when I had a conversation with coworkers?” or “Why I was always tired when I went to a place with a lot of people?”). Therefore, VR interventions for promoting self-efficacy could be designed to provide opportunities for users to investigate their emotional reactions through data reflection and to connect the insights to their real-world practices. To encourage reflection, Fleck and Fitzpatrick [44] suggest incorporating reflective questions into technology to prompt users to think about their behaviors and provide justifications or explanations for knowledge, actions, or events. Therefore, VR systems can present reflective questions such as “What about this scenario made you feel anxious?” to accompany data reflection and to provide scaffolding for individuals with autism to consider how their performance in the scenarios relates to their real-world experiences.

Finally, when reflecting on the physiological data marked during the VR simulations, the system can guide users to raise their emotional awareness by relating it to their real-world experiences. It is important for them to understand what factors trigger their emotional and physical responses, why these factors affect them in a certain way, and how they should respond to such emotions. Previous research suggests that careful observation of one’s behavior, either by themselves or by others, might be the most informative and applicable source of emotional self-awareness [45,46]. Similarly, in our study, participants with autism identified the situations where they felt anxious while reviewing the anxiety moments data and watching the recorded videos of their VR performance and described their feelings by recalling prior related experiences. Through this process, they planned more specific ways to respond to anxiety, which could be applicable to their real-world interpersonal situations.

#### Toward Data-Driven Self-Advocacy

Verbal persuasion, involving encouragement from others and self-advocacy practice, is an important source of self-efficacy because it can help individuals shape self-beliefs that they have the skills or knowledge to succeed in a given situation and have confidence in themselves [19,47].

Our study suggests that the data-driven reflection process could have a similar effect as verbal persuasion. Our participants with autism reported that they often received negative feedback about their behaviors from others but experienced validation of behaviors through our system, for example, when the data interface indicated that their voice volume was lower than they expected. These results show that data reflection creates an opportunity for individuals with autism to experience verbal persuasion through identifying what they can do well in VR scenarios and fostering internal motivation to apply what they learned about themselves in the real world.

Furthermore, the participants with autism wanted to advocate for their characteristics or strengths identified in the data reflection to others to resolve conflicts or difficulties in their interpersonal relationships (eg, family members’ negative comments on the behaviors of autistic individuals). This finding suggests that data reflection could help individuals with autism to advocate for themselves in their daily lives and workplace. To design systems that can effectively support self-advocacy, our findings suggest the importance of presenting data relevant to their daily lives and supporting them to use the data to reflect on their behaviors, build confidence, and foster self-advocacy.

In our study, reflective questions [48] enabled users with autism to take time to understand themselves and translate their thoughts and concerns into positive resolutions based on the data. The questions included the following: “[reviewing the anxiety moment data] Have you ever encountered a similar situation in real life? If so, why did you feel that way? How do you typically respond to stressful events that make you anxious?” Although the participants could not directly manipulate the data visualization interface, future studies are needed to uncover how the interface can be designed to engage users with autism to better reflect on their personal interests, skills, and experiences. This could be approached by visualizing data with metaphors familiar to individuals with autism and customizing the user interface to reflect users’ priorities and topics of interest [49-51].

### Limitations

Although our VR system could provide individuals with autism with opportunities to promote self-efficacy, there are several limitations. First, in our study, we only included participants with autism who are able to communicate and interact with others. This decision was made in our study because WorkplaceVR was designed to focus on a specific population with autism. However, future research should explore how VR interventions can also benefit participants with autism who have different communication abilities. To estimate anxiety moments, we used the algorithm offered by Microsoft Azure. Although our participants confirmed that the anxiety predictions were aligned with their subjective feelings (eg, anxiety and nervousness), future studies should investigate and apply more rigorous algorithms that can predict the anxiety levels of participants. In this study, we used the PSES-VR, a questionnaire written in Korean, and the IPQ, which was translated into Korean. However, neither of the 2 measures had been previously validated in the Korean population. Finally, we could not confirm whether participants’ experiences in the study would translate to their real-life situations through poststudy observations.

### Conclusions

This study investigated how the VR system promotes the self-efficacy of individuals with autism for their success at work. For this, we presented WorkplaceVR, a VR system that allows users to experience work-related social situations and data reflection of the user’s behavioral and physiological data. Through the VR experiment and data reflection, we confirmed that the VR system significantly improved the perceived self-efficacy of participants with autism. Our study results revealed that the VR system provided participants with autism with an opportunity to have a mastery experience in VR scenarios, self-awareness of their emotional states, and self-advocacy of their strengths and characteristics through data reflection. By addressing the expectations and challenges in the VR system for people with autism, these results contribute to not only supporting researchers who design the technology for autistic people but also helping individuals with autism have a successful work experience.

## Appendix

Multimedia Appendix 1 Contents of the two scales for evaluating social skills related to the perceived self-efficacy of the participants and the perceived sense of presence of our virtual reality system: (1) Perceived Self-Efficacy for VR Social Skill Training Scale and (2) iGroup Presence Questionnaire.

## Abbreviations

E4 band: Empatica E4 wristband
EDA: electrodermal activity
HMD: head-mounted display
HR: heart rate
IPQ: iGroup Presence Questionnaire
PSES-VR: Perceived Self-Efficacy for VR Social Skill Training Scale
ROI: region of interest
VR: virtual reality

## Data Availability

The data sets generated during this study are available from the corresponding author upon reasonable request.

## References

[1] Zaboski BA, Storch EA (2018). Comorbid autism spectrum disorder and anxiety disorders: a brief review. Future Neurol.

[2] Hollocks MJ, Pickles A, Howlin P, Simonoff E (2016). Dual cognitive and biological correlates of anxiety in autism spectrum disorders. J Autism Dev Disord.

[3] Robertson CE, Baron-Cohen S (2017). Sensory perception in autism. Nat Rev Neurosci.

[4] Bury SM, Flower RL, Zulla R, Nicholas DB, Hedley D (2021). Workplace social challenges experienced by employees on the autism spectrum: an international exploratory study examining employee and supervisor perspectives. J Autism Dev Disord.

[5] Hendricks D (2010). Employment and adults with autism spectrum disorders: challenges and strategies for success. J Vocat Rehabil.

[6] Lorenz T, Frischling C, Cuadros R, Heinitz K (2016). Autism and overcoming job barriers: comparing job-related barriers and possible solutions in and outside of autism-specific employment. PLoS One.

[7] Morris MR, Begel A, Wiedermann B (2015). Understanding the challenges faced by Neurodiverse software engineering employees: towards a more inclusive and productive technical workforce. Proceedings of the 17th International ACM SIGACCESS Conference on Computers & Accessibility.

[8] Riedelbauch S, Gaigg SB, Thiel T, Roessner V, Ring M (2023). Examining a model of anxiety in autistic adults. Autism.

[9] Lee EA, Black MH, Tan T, Falkmer T, Girdler S (2019). "I'm destined to ace this": work experience placement during high school for individuals with autism spectrum disorder. J Autism Dev Disord.

[10] Wehman P, Brooke V, Brooke AM, Ham W, Schall C, McDonough J, Lau S, Seward H, Avellone L (2016). Employment for adults with autism spectrum disorders: a retrospective review of a customized employment approach. Res Dev Disabil.

[11] Hedley D, Uljarević M, Cameron L, Halder S, Richdale A, Dissanayake C (2017). Employment programmes and interventions targeting adults with autism spectrum disorder: a systematic review of the literature. Autism.

[12] Adiani D, Itzkovitz A, Bian D, Katz H, Breen M, Hunt S, Swanson A, Vogus TJ, Wade J, Sarkar N (2022). Career interview readiness in virtual reality (CIRVR): a platform for simulated interview training for autistic individuals and their employers. ACM Trans Access Comput.

[13] Ke F, Moon J, Sokolikj Z (2020). Virtual reality–based social skills training for children with autism spectrum disorder. J Spec Educ Technol.

[14] Didehbani N, Allen T, Kandalaft M, Krawczyk D, Chapman S (2016). Virtual reality social cognition training for children with high functioning autism. Comput Hum Behav.

[15] Bozgeyikli L, Bozgeyikli E, Raij A, Alqasemi R, Katkoori S, Dubey R (2017). Vocational rehabilitation of individuals with autism spectrum disorder with virtual reality. ACM Trans Access Comput.

[16] Bozgeyikli LL, Bozgeyikli E, Katkoori S, Raij A, Alqasemi R (2018). Effects of virtual reality properties on user experience of individuals with autism. ACM Trans Access Comput.

[17] Smith MJ, Ginger EJ, Wright K, Wright MA, Taylor JL, Humm LB, Olsen DE, Bell MD, Fleming MF (2014). Virtual reality job interview training in adults with autism spectrum disorder. J Autism Dev Disord.

[18] Mesa-Gresa P, Gil-Gómez H, Lozano-Quilis JA, Gil-Gómez JA (2018). Effectiveness of virtual reality for children and adolescents with autism spectrum disorder: an evidence-based systematic review. Sensors (Basel).

[19] Bandura A (1977). Self-efficacy: toward a unifying theory of behavioral change. Psychol Rev.

[20] Bandura A (2007). Self-efficacy conception of anxiety. Anxiety Res.

[21] Ward DM, Esposito MC (2018). Virtual reality in transition program for adults with autism: self-efficacy, confidence, and interview skills. Contemp Sch Psychol.

[22] Spiel K, Frauenberger C, Keyes O, Fitzpatrick G (2019). Agency of autistic children in technology research—a critical literature review. ACM Trans Comput Hum Interact.

[23] Leadbitter K, Buckle KL, Ellis C, Dekker M (2021). Autistic self-advocacy and the neurodiversity movement: implications for autism early intervention research and practice. Front Psychol.

[24] Kim JG, Kim T, Kim SI, Jang SY, Lee EB, Yoo H, Han K, Hong H (2022). The workplace playbook VR: exploring the design space of virtual reality to foster understanding of and support for autistic people. Proc ACM Hum Comput Interact.

[25] VIVE Pro 2. VIVE.

[26] Yoo HJ, Bahn G, Cho IH, Kim EK, Kim JH, Min JW, Lee WH, Seo JS, Jun SS, Bong G, Cho S, Shin MS, Kim BN, Kim JW, Park S, Laugeson EA (2014). A randomized controlled trial of the Korean version of the PEERS(®) parent-assisted social skills training program for teens with ASD. Autism Res.

[27] Laugeson EA, Frankel F, Gantman A, Dillon AR, Mogil C (2012). Evidence-based social skills training for adolescents with autism spectrum disorders: the UCLA PEERS program. J Autism Dev Disord.

[28] In-cafe training guide. Stone Creek Coffee Factory.

[29] (2019). Service and training manual. Smith Coffee and Cafe.

[30] Ding D, Brinkman WP, Neerincx MA (2020). Simulated thoughts in virtual reality for negotiation training enhance self-efficacy and knowledge. Int J Hum Comput Stud.

[31] Huang Y, Richter E, Kleickmann T, Richter D (2022). Comparing video and virtual reality as tools for fostering interest and self-efficacy in classroom management: results of a pre-registered experiment. Br J Educ Technol.

[32] Bandura A, Schwarzer R (1992). Self-efficacy mechanism in psychobiologic functioning. Self-Efficacy: Thought Control of Action.

[33] Simões M, Bernardes M, Barros F, Castelo-Branco M (2018). Virtual travel training for autism spectrum disorder: proof-of-concept interventional study. JMIR Serious Games.

[34] Flask homepage. Flask.

[35] Machine learning algorithms. Microsoft Azure.

[36] Tobii G2oM. Tobii.

[37] Bandura A (2011). On the functional properties of perceived self-efficacy revisited. J Manage.

[38] Sung C, Connor A, Chen J, Lin C-C, Kuo H-J, Chun J (2019). Development, feasibility, and preliminary efficacy of an employment-related social skills intervention for young adults with high-functioning autism. Autism.

[39] Pedrazza M, Trifiletti E, Berlanda S, Bernardo GA (2013). Self-efficacy in social work: development and initial validation of the self-efficacy scale for social workers. Soc Sci.

[40] Regenbrecht H, Schubert T (2002). Real and illusory interactions enhance presence in virtual environments. Presence Teleoperators Virtual Environ.

[41] Braun V, Clarke V (2006). Using thematic analysis in psychology. Qual Res Psychol.

[42] Nguyen W, Ownsworth T, Nicol C, Zimmerman D (2020). How I see and feel about myself: domain-specific self-concept and self-esteem in autistic adults. Front Psychol.

[43] Lamash L, Meyer S (2022). Work-related self-efficacy and illness identity in adults with autism. Int J Environ Res Public Health.

[44] Fleck R, Fitzpatrick G (2010). Reflecting on reflection: framing a design landscape. Proceedings of the 22nd Conference of the Computer-Human Interaction Special Interest Group of Australia on Computer-Human Interaction.

[45] Mazefsky CA, Herrington J, Siegel M, Scarpa A, Maddox BB, Scahill L, White SW (2013). The role of emotion regulation in autism spectrum disorder. J Am Acad Child Adolesc Psychiatry.

[46] Conner CM, White SW, Beck KB, Golt J, Smith IC, Mazefsky CA (2019). Improving emotion regulation ability in autism: the emotional awareness and skills enhancement (EASE) program. Autism.

[47] Samson A, Solmon M (2011). Examining the sources of self-efficacy for physical activity within the sport and exercise domains. Int Rev Sport Exerc Psychol.

[48] Fleck R, Fitzpatrick G (2009). Teachers’ and tutors’ social reflection around SenseCam images. Int J Hum Comput Stud.

[49] Choe EK, Lee B, Zhu H, Riche NH, Baur D (2017). Understanding self-reflection: how people reflect on personal data through visual data exploration. Proceedings of the 11th EAI International Conference on Pervasive Computing Technologies for Healthcare.

[50] Cho J, Xu T, Zimmermann-Niefield A, Voida S (2022). Reflection in theory and reflection in practice: an exploration of the gaps in reflection support among personal informatics apps. Proceedings of the 2022 CHI Conference on Human Factors in Computing Systems.

[51] Ayobi A, Marshall P, Cox AL (2020). Trackly: a customisable and pictorial self-tracking app to support agency in multiple sclerosis self-care. Proceedings of the 2020 CHI Conference on Human Factors in Computing Systems.

