# Supplementary material for: Promoting Self-Efficacy of Individuals With Autism in Practicing Social Skills in the Workplace Using Virtual Reality and Physiological Sensors: Mixed Methods Study
Source: JMIR Form Res. 2024 Jan 11;8:e52157. doi: 10.2196/52157 (PMC10811570; doi:10.2196/52157)
Supplement: Multimedia Appendix 1 [file formative_v8i1e52157_app1.docx]

**Multimedia Appendix 1.**

**Perceived Self-efficacy for VR social skill training scale**

※ The following questions related to social skills in the WorkplaceVR program. After reading each item below, please honestly check the item the five items provided.

|  | Question | Very unsure | Unsure | Neutral | Confident | Very Confident |
| --- | --- | --- | --- | --- | --- | --- |
| 1 | I can clearly express my intentions or thoughts to others. | 1 | 2 | 3 | 4 | 5 |
| 2 | I can listen attentively to what others are saying. | 1 | 2 | 3 | 4 | 5 |
| 3 | When conversing with others, I can start speaking after they finish. | 1 | 2 | 3 | 4 | 5 |
| 4 | Even when flustered during a conversation, I can provide appropriate responses or guidance. | 1 | 2 | 3 | 4 | 5 |
| 5 | I can speak to others in a clear voice that is neither too loud nor too soft. | 1 | 2 | 3 | 4 | 5 |
| 6 | I can maintain an appropriate distance when talking to others. | 1 | 2 | 3 | 4 | 5 |
| 7 | I can engage in a conversation while looking into the other person's eyes. | 1 | 2 | 3 | 4 | 5 |
| 8 | I can effectively follow given instructions. | 1 | 2 | 3 | 4 | 5 |

**iGroup Presence Questionnaire**

※ The following items are related to how closely the WorkplaceVR program simulates the real workplace environment. A higher score indicates 'fully agree,' while a lower score indicates 'fully disagree.' After reading each item below, please honestly check the item among the seven items provided.

|  | Question | Fully disagree ⬄ Fully agree | | | | | | |
| --- | --- | --- | --- | --- | --- | --- | --- | --- |
| 1 | In the computer generated world I had a sense of "being there” | -3 | -2 | -1 | 0 | +1 | +2 | +3 |
| 2 | Somehow I felt that the virtual world surrounded me. | -3 | -2 | -1 | 0 | +1 | +2 | +3 |
| 3 | I felt like I was just perceiving pictures. | -3 | -2 | -1 | 0 | +1 | +2 | +3 |
| 4 | I did not feel present in the virtual space. | -3 | -2 | -1 | 0 | +1 | +2 | +3 |
| 5 | I had a sense of acting in the virtual space, rather than operating something from outside. | -3 | -2 | -1 | 0 | +1 | +2 | +3 |
| 6 | I felt present in the virtual space. | -3 | -2 | -1 | 0 | +1 | +2 | +3 |
| 7 | How aware were you of the real world surrounding while navigating in the virtual world? (i.e. sounds, room temperature, other people, etc.)? | -3 | -2 | -1 | 0 | +1 | +2 | +3 |
| 8 | I was not aware of my real environment. | -3 | -2 | -1 | 0 | +1 | +2 | +3 |
| 9 | I still paid attention to the real environment. | -3 | -2 | -1 | 0 | +1 | +2 | +3 |
| 10 | I was completely captivated by the virtual world. | -3 | -2 | -1 | 0 | +1 | +2 | +3 |
| 11 | How real did the virtual world seem to you? | -3 | -2 | -1 | 0 | +1 | +2 | +3 |
| 12 | How much did your experience in the virtual environment seem consistent with your real world experience ? | -3 | -2 | -1 | 0 | +1 | +2 | +3 |
| 13 | How real did the virtual world seem to you? | -3 | -2 | -1 | 0 | +1 | +2 | +3 |
| 14 | The virtual world seemed more realistic than the real world. | -3 | -2 | -1 | 0 | +1 | +2 | +3 |
